# Retraction Note: An experimental investigation of unique high stepup boost converter for electric vehicle and solar photovoltaic

**DOI:** 10.1038/s41598-026-55808-y

**Published:** 2026-06-03

**Authors:** T. Mariprasath, Sujata Shivashimpiger, Marco Rivera, Patrick Wheeler, M. Pala Prasad Reddy, Shaik Muqthiar Ali, Venkatesh Peruthambi, Jakson Bonaldo

**Affiliations:** 1 K.S.R.M College of Engineering (Autonomous), Kadapa, Andhra Pradesh 516005 India; 2 Nitte Meenakshi Institute of Technology (NMIT), Nitte (Deemed to be University), Bangalore, India; 3 Power Electronics, Machines and Control (PEMC) Research Institute, Department of Electrical and Electronic Engineering, Faculty of Engineering, University of Nottingham, Nottingham, NG7 2GT UK; 4 Department of Electrical and Electronics Engineering, Institute of Aeronautical Engineering, Hyderabad, Telangana 500043 India; 5 Annamacharaya University, Rajampet, Andhra Pradesh India; 6 Electrical and Electronics Engineering, Mohan Babu University, Tirupati, Andhra Pradesh India; 7 Department of Electrical Engineering, Federal University of Mato Grosso, Avenida Fernando Correa da Costa, 1367, Cuiaba, 78060-900 Brazil

Retraction of: *Scientific Reports* 10.1038/s41598-025-25807-6, published online 19 January 2026

The Editors have retracted this Article.

Following publication, concerns were raised about the rationale for the approach presented, the analyses and the claims in the Article. A post-publication review of the Authors’ methods and analyses revealed a lack of clarity in the presented Figures and in their related descriptions as well as in the reported equations. The main concerns are:

• the converter is not a boost converter but instead has a DC-DC Push-Pull converter topology;

• the presence of a 12 V linear regulator connected in series with the PV panels in Figure 2a and 2b;

• the incorrect polarity of the diodes on the secondary side of the transformer in Figure 2a and 2b;

• the inversion of the polarity of diodes D1 and D2 in Figure 2a and 2b;

• diodes D1 and D2 in the gate-drive circuit are oriented in a way that would hinder the intended fast turn-off behaviour in Figure 2a and 2b;

• pin 15 of the SG3525 integrated circuit is not connected to a power supply (VCC) in Figure 2a and 2b;

• Figure 2c presents rectified sinusoidal waveforms that do not correspond to any signals existing in the proposed circuit;

• the transfer function in equation 11 is mathematically incomplete because it neglects the effect of the inductances.

The Editors therefore no longer have confidence in the reliability of the content of this Article.

Marco Rivera, Patrick Wheeler, and Jakson Bonaldo agree with the retraction and its wording. T. Mariprasath did not explicitly state if they agree or disagree with this retraction. Sujata Shivashimpiger, M. Pala Prasad Reddy, Shaik Muqthiar Ali, and Venkatesh Peruthambi did not respond to the correspondence from the Editors about this retraction.

